# The Impact of Complex PTSD on Suicide Risk in Patients with Bipolar Disorder: A Cross-Sectional Study

**DOI:** 10.3390/jcm13030673

**Published:** 2024-01-24

**Authors:** Anna Maria Iazzolino, Marta Valenza, Martina D’Angelo, Grazia Longobardi, Valeria Di Stefano, Giulia Visalli, Luca Steardo, Caterina Scuderi, Luca Steardo

**Affiliations:** 1 Psychiatry Unit, Department of Health Sciences, University of Catanzaro Magna Graecia, 88100 Catanzaro, Italy; martina.dangelo001@studenti.unicz.it (M.D.); grazia.longobardi@studenti.unicz.it (G.L.); valeria.distefano@studenti.unicz.it (V.D.S.); giulia.visalli@studenti.unicz.it (G.V.); steardo@unicz.it (L.S.J.); 2 Department of Physiology and Pharmacology “Vittorio Erspamer”, Sapienza University of Rome, 00185 Rome, Italy; marta.valenza@uniroma1.it (M.V.); luca.steardo@uniroma1.it (L.S.); caterina.scuderi@uniroma1.it (C.S.); 3 University Giustino Fortunato, 82100 Benevento, Italy

**Keywords:** suicide, cPTSD, trauma, bipolar disorder

## Abstract

Background: Patients with bipolar disorder (BD) are more likely than the general population to experience traumatic events, particularly during childhood, and these may predict and be a risk factor for the development of complex PTSD (cPTSD). The presence of multiple traumas plays a relevant role from a psychopathological point of view, but little is known about the effect this may have on suicide attempts in patients with BD. Methods: A cross-sectional study was conducted comparing socio-demographic and clinical characteristics, recruiting 344 patients diagnosed with BD I and II, screened for the presence (or absence) of cPTSD using the International Trauma Questionnaire (ITQ). Suicide attempts were assessed directly during the clinical interview and from the patient’s medical record. Results: The results emerging from the study indicate that cPTSD can be considered a risk factor for suicide attempts in patients with BD. Furthermore, evidence is provided to support the idea that cPTSD is highly prevalent in patients with BD and is related to a higher psychopathological burden. Conclusions: The results recommend an urgent and comprehensive assessment of suicidal risk in patients with comorbidity of both bipolar disorder and cPTSD. There is a crucial demand for early intervention initiatives and proactive prevention strategies to address the intricate intersection of these mental health challenges.

## 1. Introduction

The recent inclusion of complex post-traumatic stress disorder (cPTSD) as a new and autonomous diagnostic category in the International Classification of Diseases, 11th Edition (ICD-11), has brought attention to this recently classified mental health disorder. This recognition not only acknowledges the significance of complex traumatic stress disorder but also paves the way for extensive research and exploration into this lately categorized condition [1,2].

Complex post-traumatic stress disorder is a grievous and disabling mental disorder that arises in response to multifaceted, prolonged, and/or inescapable traumatic life events [3,4]. Indeed, patients suffering from cPTSD commonly report continued exposures to different injuries including, but not limited to, domestic or community physical or psychological violence [3]. Its diagnostic six-factor structure, consisting of six different symptom clusters, includes three core PTSD criteria (i.e., reexperiencing, avoidance, and hypervigilance), along with three new ones (i.e., chronic and pervasive disturbances of self-organization (DSO) symptoms defined as emotional dysregulation, interpersonal difficulties, and negative self-reputation) [1,3]. cPTSD presents a prevalence in the general population ranging between 1 and 8% worldwide, but its frequency rate easily rises up to 50% in mental health facilities [3], greatly increasing the chances of comorbidity with other psychiatric disorders, including anxiety disorders, major depressive disorder, bipolar disorder (BD), personality disorders, and suicidality [5,6].

In this context, it is important to recognize that BD shares with cPTSD several features: the multifactorial etiopathogenesis, the complex role played by trauma, the worsening of the quality of life, the prolonged course of the disorder, and the significant increase in suicidal risk [7]. Moreover, similarly to cPTSD, BD is a common severe condition in psychiatry primary care, and there is a growing body of literature demonstrating the effectiveness of a combined intervention to treat this comorbidity [8,9]. As noted, suicidal behavior is very frequent among subjects with BD, as up to 4–19% of them attempt suicide to end their life, while 20–60% of them attempt suicide at least once in their lifetime [10]. The risk of suicide attempt and suicide death is up to 10–30 times higher in patients with BD than in the general population [11]. In this scenario, C-PTSD seems to be a further relevant risk factor for suicidal attempts [12].

Keeping in mind the high disease burden that complex post-traumatic stress disorder and bipolar disorder individually produce, their comorbidity with the consequent worsening of the prognosis suggests a careful assessment of suicidal risk, especially in light of the absence of investigations in this specific area [13,14,15].

The current study aimed to examine the relationship between cPTSD, BD type I and type II, and suicidality in a primary mental health care setting for outpatients suffering from these complex disorders condition.

## 2. Materials and Methods

### 2.1. Study Design, Recruitment of Participant, and Eligibility Criteria

For this cross-sectional study, subjects were consecutively recruited at the Psychiatric Unit of the University Hospital Mater Domini of Catanzaro (Italy) from January 2021 until December 2023. Three hundred forty-four outpatients fulfilled the following inclusion criteria: age ≥ 18 and <65 years, diagnosis of BD according to the Diagnostic and Statistical Manual of Mental Disorders 5th edition (DSM-5) [16], and willingness to participate in the study. Subjects with inability to give written consent to participate in the study, moderate or severe cognitive impairment evaluated with Mini-Mental State Evaluation (MMSE) (cut-off > 25), or comorbidity with any medical disease that could affect the psychopathological condition were considered not eligible and excluded. A written informed consent was collected for each participant after a full description of the study aims and design was provided. The study was approved by the Ethics Committee of the University of Catanzaro (307/2020) on 25 November 2020 and it was carried out following the latest version of the Declaration of Helsinki. Socio-demographic and clinical characteristics, including diagnosis, familiarity with psychiatric disorders, age at onset of the disorder, number of episodes (depressive, manic, hypomanic), hospitalizations, seasonality, lifetime suicide attempts, mixed features according to the DSM-5 specifier, psychotic symptoms, aggressive behavior, and antidepressant-induced mania were documented with an ad-hoc schedule. Age at onset was retrospectively assessed as age at first affective episode, either depressive or hypomanic/manic.

#### Psychometric Tool

The Structured Interview for DSM-5 Disorders, Clinician Version (SCID-5-CV) [17], was used by trained psychiatrists to assess the diagnosis. The International Trauma Questionnaire (ITQ) [18] was used to assess experienced trauma and evaluate the presence of cPTSD consistent with the organizing principles of the ICD-11. This tool is an 18-question self-report measure made up of two major subscales, the PTSD and the DSO. Participants were asked to indicate how much they have been bothered by six core PTSD symptoms in the past month using a five-point Likert scale ranging from 0 (‘Not at all’) to 4 (‘Extremely’). In the PTSD subscale, two symptoms are related to the re-experiencing cluster of symptoms; two symptoms to the avoidance cluster; and two to the sense of current hyperarousal. A set of six questions detect the DSO symptoms related to the three clusters: “affective dysregulation”; “negative self-concept”; and “disturbed relationships”. This is important to recognize and assess a probable cPTSD. A probable PTSD diagnosis was made if a trauma was reported, a score of ≥2 (‘Moderately’) was obtained for at least one of two symptoms from each cluster, indicating the frequency of symptoms in the previous 2 weeks on a four-point Likert-scale, ranging from 0 (not at all) to 3 (nearly every day). Total scores can range from 0 to 27. 

A probable cPTSD diagnosis was considered if criteria for PTSD were met, in addition to scoring ≥2 (‘Moderately’) for at least one symptom from each of the three DSO symptom clusters. Suicidality was assessed directly during the clinical interview and from the patient’s record. We considered only the attempted suicide and not suicidal ideation. As the variable ‘suicide attempt,’ we considered patients with a history of unsuccessful suicide attempts that led to hospitalization, underscoring the severity of the attempt.

### 2.2. Statistical Analysis

Statistical analysis was executed by using the Statistical Package for Social Sciences Version 26 (SPSS, Chicago, IL, USA). Descriptive analyses were carried out to evaluate the distribution of the variables in the whole sample and the normality distribution was assessed through the normality Shapiro–Wilk test. The results were reported as frequencies (for categorical variables), or as mean ± standard deviation (SD) or median and interquartile range (IQR) (for continuous variables) according to the normality of the distribution. The sample was s divided into two groups according to the presence/absence of cPTSD. Chi-square test, Student’s t-test, or Mann–Whitney U test bivariate analyses were run (as appropriate) to test the difference between socio-demographics and tool scores between groups. Suicide variable in the dichotomous form was used to test the strength of association with the risk of suicide attempt and the odds ratio with 95% confidence interval was calculated. A logistic regression model was used to describe the association between the presence of cPTSD as a dependent variable, and suicide attempts as an independent variable. The level of statistical significance was set at a value of *p* ≤ 0.05.

## 3. Results

### 3.1. Psychopathological Characteristics of the Sample

As shown in Table 1, 344 patients who met the inclusion criteria were included in the study, with BD-I being the most common diagnosis (73.5%, N = 253; BD-II: 26.5%, N = 91). The mean age was 46.88 (±13.94) years, and a significant percentage of participants were female (51.5%). Many participants were married (54.1%), college graduates (71.5%), and employed (49.7%). About 36% (N = 124) reported having experienced abuse in their lifetime. Clinical characteristics included a mean age at BD onset of 25.73 (±9.40) years, with a mean duration of untreated disease of 5.84 (±10.31) years. Participants experienced a mean of 5.81 (±5.92) depressive episodes, 4.04 (±4.41) manic episodes, and 2.65 (±2.69) hypomanic episodes. The mean number of affective episodes was 11.39 (±9.87). Most patients had aggressive behaviors (56.4%), mixed features (56.1%), and psychotic symptoms (41.9%). Seasonality was common (49.7%), as was antidepressant switching (24.7%) and a history of suicide attempts (40.1%).

### 3.2. Differences between cPTSD and Non-cPTSD Groups

Regarding the Impact of Events Scale (ITQ) scores, 44.8% of the enrolled patients (N = 154) met the criteria for the diagnosis of complex post-traumatic stress disorder (cPTSD). Patients with cPTSD were generally younger, more often male graduates, and with a family history of psychiatric disorders and more lifetime abuses. Most of them were diagnosed with bipolar disorder I, had an earlier age at onset, shorter duration of untreated illness, more depressive and manic episodes, more antidepressant switches, aggressive behaviors, mixed features, psychotic symptoms, and seasonality. About 71.4% of patients with cPTSD reported suicide attempts (*p* < 0.001). Among these, 53.9% had at least one attempt, 18.8% had at least one hospitalization, and 35.8% had more than two attempts. Differences in scores at the ITQ were significant, with DSO and PTSD scores higher in the cPTSD group (all *p* values < 0.001) (Table 2).

### 3.3. Estimation of Suicide Risk

We tested the strength of association with suicide attempt for the identification of cPTSD as risk factors by odds ratio (OR) with a 95% confidence interval (CI). Patients with cPTSD were found to be at a significantly higher risk for attempting suicide. Odds ratios were 14.46 (95% CI 8.50–24.63, *p* < *0*.001) (Table 3).

### 3.4. Logistic Regression

Moreover, at the logistic regression, a significative association was found between cPTSD and suicide (*p* < 0.001) (Table 4).

## 4. Discussion

This study represents the first to demonstrate the prevalence of cPTSD in individuals with BD, as well as to elucidate the connection between suicide and cPTSD within these populations. It is well known that the latest disorder has a 1–8% prevalence in the general population and up to 50% prevalence in mental health facilities [3]. After the cPTSD inclusion in the ICD-11, growing evidence demonstrated the higher prevalence of cPTSD in different populations: veterans, war captives, firefighters, police officers, refugees [19,20,21,22,23,24], and in some psychiatric disorders such as personality disorders [1,13]. The present study strongly supports the notion that cPTSD is highly prevalent even in patients suffering from BD. In the subjects investigated those with cPTSD were younger, mostly male, and reported more lifetime abuses. The latter aspect was mostly investigated in the literature. Numerous studies exploring traumatic events highlighted their strong association with later BD development [25,26,27]. This association is particularly evident for emotional abuse, maltreatment, bullying, and parental loss [25,26,27]. On the other hand, growing evidence has demonstrated that patients diagnosed with PTSD report a higher number of traumatic experiences, emotional neglect in particular [28], and that adverse childhood experiences (e.g., physical/emotional neglect [29]) can predict and be a risk factor for cPTSD development [30]. Additionally, adverse childhood experiences were associated with clinical severity regardless of diagnosis [31]. Exposure to trauma in patients with complex post-traumatic stress disorder (cPTSD) has been associated with psychotic symptoms [29], heightened levels of psychopathological comorbidities, and diminished psychological well-being [32]. Regarding bipolar disorder, trauma exposure may contribute to the exacerbation of bipolar symptoms. Research suggests that individuals with a history of trauma may experience dissociation that is closely linked to the onset of psychotic symptoms [15]. According to this evidence, patients in our sample with cPTSD met the diagnostic criteria for BD-I, with more severe psychopathology (i.e., earlier age at onset, a higher number of depressive and manic episodes, more antidepressant switches, aggressive behaviors, mixed features, psychotic symptoms, and seasonality). Thus, we may hypothesize that childhood trauma carries out an additive effect on the development of both conditions leading to a more psychopathological impairment.

Of greater importance is that patients with cPTSD, studied in the present investigation, reported previous suicide attempts. This result may be explained considering that firstly, patients with BD who endorsed prior suicide attempts exhibited significantly higher rates of trauma exposure than those with BD who negated a history of suicide attempts [33]; secondly, patients with cPTSD reported higher depression, anxiety, and self-harm compared to PTSD [34]. This result is strongly reinforced by the evidence that cPTSD can be considered a risk factor for suicide attempts in patients with BD. Moreover, at the logistic regression, a significative association was found between cPTSD and suicide. 

A possible explanation of these findings may be in the complex interplay between traumatic experiences, alienation feelings or dissociative symptoms, consequent loneliness, and low self-esteem that may act on the three core post-traumatic DSO symptoms. 

Research reported that patients with cPTSD described high levels of dissociation, anxiety, anger, more emotional overexcitability, social isolation, and sleep difficulties [24], leading to a less adaptive emotion-oriented coping strategy under stress and impaired functioning [35]. In this regard, childhood traumatic experiences are linked to the development of maladaptive negative cognitive styles that may evolve in cPTSD [36]. On the other hand, studies identified significantly higher levels of dissociation in cPTSD [37]. Notably, dissociative phenomena are also reported in BD, especially in BD-I, and are closely related to psychopathology (i.e., psychotic symptoms and previous suicide attempts) [15]. In patients with BD and cPTSD, therefore, dissociation mediates the relationship between reported trauma exposure during childhood and cPTSD and DSO symptoms [38], contributing to a high psychopathological impairment and poor outcome when the two conditions co-exist. As a result, individuals who are exposed to repeated, prolonged trauma and multiple forms of trauma (i.e., complex trauma) frequently experience feelings of emptiness, loneliness, disconnection, and alienation from close relationships and society, contributing to the self-concept and relational difficulties that are characteristic of cPTSD [39,40,41,42]. Loneliness was strongly related to symptoms of cPTSD, and emotional and social loneliness was linked to DSO symptoms, playing an important role in the development of cPTSD [23,43]. 

Our hypothesis should be interpreted in the context of recent literature, taking into consideration the reciprocal nature of the relationship. Symptoms associated with complex PTSD (cPTSD) may not only be a consequence of adverse experiences but could also contribute to the development of feelings such as loneliness, depression, anxiety, and low self-esteem, along with Dysregulation of Self and Others (DSO) symptoms. Moreover, these cPTSD symptoms may exacerbate difficulties in maintaining close relationships, leading to heightened experiences of loneliness, alienation, and dissociation. Future studies are warranted to explore the potential unidirectional or bidirectional nature of this relationship, shedding light on the complex interplay between cPTSD symptoms and their impact on mental well-being and interpersonal dynamics [39]. Although the directionality of the relationship remains uncertain, we hypothesize that this complex interplay may be a risk factor for suicide attempts. It is notable to highlight that dissociation has been linked to non-suicidal self-injury [44,45], and an unpleasant feeling of a lack of connection to others, loneliness, and objective social isolation were significantly associated with suicidal behavior in patients with psychiatric disorders [46], and as such are included in the risk assessment of suicide [47].

Therefore, it is important for clinicians working with BD to routinely screen for cPTSD diagnosis by investigating traumatic experiences, dissociative experiences, and loneliness feelings to detect the earlier potential risk of self-injury and or suicide attempts [38]. In this regard, our results have implications for differential diagnosis and for the development of targeted treatments for cPTSD. Meta-analytic findings [48] suggest that standard PTSD interventions may be effective in reducing symptoms of cPTSD. Nevertheless, cPTSD is a more debilitating condition than PTSD and there is a need to test the effectiveness of new and existing interventions to improve symptoms [48,49]. 

The investigation of cPTSD in patients with BD may potentially facilitate access to more tailored treatment interventions, as well as contribute to an increased research focus on disorders specifically associated with stress [49].

## 5. Limitations

The study presents some limitations. First, this study involved a cross-section of a large proportion of the psychiatric population, which prevents establishing definite causal relationships. Second, the retrospective nature of the study about suicide attempts and trauma could be affected by recall bias regarding the assembly and reliability of memories. Third, the type of and cumulative child abuse were not assessed. Despite these limitations, this was the first attempt to evaluate the prevalence of cPTSD in a large and real-world sample of patients with BD and to shed light on the role and implications of suicide attempts in this population. However, to further deepen our understanding of this relationship, future work could build on these findings by using a longitudinal design to explore the role of cPTSD symptoms as they develop over time and their impact on the risk of suicide attempts.

## 6. Conclusions

cPTSD is frequently comorbid with bipolar disorder (BD), and this overlap is strongly associated with an increased risk of suicide. Therefore, a comprehensive assessment of suicide risk is crucial during all clinical visits for patients with both BD and cPTSD. This clinical evaluation should encompass a thorough examination of the mental state, a careful exploration of traumatic experiences, and a comprehensive review of current suicidal intentions and past suicide attempts. Early intervention programs and prevention strategies are imperative. Additionally, integrating therapeutic programs that can complement the treatment of bipolar disorder with innovative psychotherapeutic approaches, such as Eye Movement Desensitization and Reprocessing (EMDR) [50], cognitive processing therapy (CPT), or dialectical [51], may prove beneficial.

## Figures and Tables

**Table 1:** Main characteristics in patients with cPTSD vs. without cPTSD.

	cPTSD		Statistic
Characteristics	No N = 190	Yes N = 154	*p*
Age M, (SD±)	48.7 (±13.48)	44.65 (±14.21)	0.008
Age at onset M, (SD±)	27.02 (±10.29)	24.15 (±7.93)	0.001
duration of untreated illness M, (SD±)	7.03 (±10.98)	4.36 (±9.25)	0.087
number of depressive episodes M, (SD±)	5.71 (±6.84)	5.94 (±4.56)	0.002
number of manic episodes M, (SD±)	3.94 (±5.60)	4.12 (±3.12)	0.005
number of hypomanic episodes M, (SD±)	2.50 (±2.12)	2.84 (±3.27)	0.658
numbers of episodes M, (SD±)	10.45 (±10.85)	12.56 (±8.40)	<0.001
Female N (yes%)	108 (56.8%)	69 (44.8%)	0.026
Graduation N (yes%)	127 (66.8%)	119 (77.3%)	0.033
Marital status N (yes%)	93 (48.9%)	93 (60.4%)	0.107
Employed N (yes%)	96 (50.5%)	75 (48.7%)	0.938
Diagnosis of bipolar disorder I N (yes%)	115 (60.5%)	138 (89.6%)	<0.001
Diagnosis of bipolar disorder II N (yes%)	75 (39.5%)	16 (19.4%)	<0.001
Family history of psychiatric disorder N (yes%)	91 (47.9%)	99 (64.3%)	0.002
Seasonality N (yes%)	78 (41.9%)	93 (60.8%)	<0.001
Aggressive behaviors N (yes%)	83 (43.7%)	111 (72.1%)	<0.001
Mixed features N (yes%)	71 (37.4%)	122 (79.2%)	<0.001
Lifetime abuse N (yes%)	45 (23.7%)	79 (51.3%)	<0.001
Psychotic symptoms N (yes%)	14 (7.5%)	130 (84.4%)	<0.001
Antidepressant mania N (yes%)	20 (10.5%)	65 (42.2%)	<0.001
Suicide attempts N (yes%)	28 (14.7%)	110 (71.4%)	<0.001

**Table 2:** ITQ scores between groups.

cPTSD									
		No N = 190				Yes N = 154			
		Mean	SD	Median	IQR	Mean	SD	Median	IQR
PTSD scores	Re-experiencing	0.3	0.47	0	1	2.93	0.65	3	0
	Avoidance	0.15	0.36	0	0	2.7	0.5	3	1
	Hyperarousal	0.34	0.47	0	1	2.83	0.55	3	0
DSO scores	Affective dysregulation	0.30	0.48	0	1	3.47	0.54	3	0
	Negative self-concept	0.09	0.29	0	0	2.83	0.67	3	1
	Disturbances in relationships	0.28	0.59	0	0	3.44	0.59	3	1

DSO, Disturbances in Self-Organization; PTSD, Post-traumatic stress disorder.

**Table 3:** Risk estimation of suicide in cPTSD sample.

		95% Confidence Intervals		
	Odds Ratio	Lower	Upper	*p*
Suicide attempts	14.464	8.496	24.626	
Fisher’s exact test	14.316	8.237	25.590	<0.001

**Table 4:** Logistic regression model.

			Wald Test			
	Estimate	Standard Error	z	Wald Statistic	df	*p*
(Intercept)	−1.303	0.170	−7.667	58.784	1	<0.001
Suicide attempts	2.672	0.271	9.841	96.844	1	<0.001

Note. cPTSD level ‘1’ coded as class 1.

## Data Availability

The data that support the findings of this study are available from the corresponding author, upon reasonable request.
